# Internal Validation of the Predictive Performance of Models Based on Three ED and ICU Scoring Systems to Predict Inhospital Mortality for Intensive Care Patients Referred from the Emergency Department

**DOI:** 10.1155/2022/3964063

**Published:** 2022-04-25

**Authors:** Zahra Rahmatinejad, Benyamin Hoseini, Fatemeh Rahmatinejad, Ameen Abu-Hanna, Robert Bergquist, Ali Pourmand, MirMohammad Miri, Saeid Eslami

**Affiliations:** ^1^Department of Medical Informatics, Faculty of Medicine, Mashhad University of Medical Sciences, Mashhad, Iran; ^2^Pharmaceutical Research Center, Mashhad University of Medical Sciences, Mashhad, Iran; ^3^Department of Health Information Technology, Faculty of Paramedical Sciences, Mashhad University of Medical Sciences, Mashhad, Iran; ^4^Department of Medical Informatics, Amsterdam UMC-Location AMC, University of Amsterdam, Netherlands; ^5^Ingerod, SE-454 94 Brastad, Sweden; Formerly UNICEF/UNDP/World Bank/WHO Special Programme for Research and Training in Tropical Diseases (TDR), World Health Organization, Geneva, Switzerland; ^6^Department of Emergency Medicine, The George Washington University, School of Medicine and Health Sciences, Washington, DC, USA; ^7^Department of Critical Care and Anesthesiology, Imam Hossein Hospital, Shahid Beheshti University of Medical Sciences, Tehran, Iran

## Abstract

**Background:**

A variety of scoring systems have been introduced for use in both the emergency department (ED) such as WPS, REMS, and MEWS and the intensive care unit (ICU) such as APACHE II, SAPS II, and SOFA for risk stratification and mortality prediction. However, the performance of these models in the ICU remains unclear and we aimed to evaluate and compare their performance in the ICU.

**Methods:**

This multicenter retrospective cohort study was conducted on severely ill patients admitted to the ICU directly from the ED in seven tertiary hospitals in Iran from August 2018 to August 2020. We evaluated all models in terms of discrimination (AUROC), the balance between positive predictive value and sensitivity (AUPRC), calibration (Hosmer-Lemeshow test and calibration plots), and overall performance using the Brier score (BS). The endpoint was considered inhospital mortality.

**Results:**

Among the 3,455 patients included in the study, 54.4% of individuals were male (*N* = 1,879) and 26.5% deceased (*N* = 916). The BS for the WPS, REMS, MEWS, APACHE II, SAPS II, and SOFA were 0.178, 0.165, 0.183, 0.157, 0.170, and 0.182, respectively. The AUROC of these models were 0.728 (0.71-0.75), 0.761 (0.74-0.78), 0.682 (0.66-0.70), 0.810 (0.79-0.83), 0.767 (0.75-0.79), and 0.785 (0.77-0.80), respectively. The AUPRC was 0.517 (0.50-0.53) for WPS, 0.547 (0.53-0.56) for REMS, 0.445 (0.42-0.46) for MEWS, 0.630 (0.61-0.65) for APACHE II, 0.559 (0.54-0.58) for SAPS II, and 0.564 (0.54-0.57) for SOFA. All models except the MEWS and SOFA had good calibration. The most accurate model belonged to APACHE II with lowest BS.

**Conclusion:**

The APACHE II outperformed all the ED and ICU models and was found to be the most appropriate model in predicting inhospital mortality of patients in the ICU in terms of discrimination, calibration, and accuracy of predicted probability. Except for MEWS, the rest of the models had fair discrimination and partially good calibration. Interestingly, although the REMS is less complicated than the SAPS II, both models exhibited similar performance. Clinicians can utilize the REMS as part of a larger clinical assessment to manage patients more effectively.

## 1. Introduction

An important responsibility of clinicians in acute medical units is making tough decisions about the provision of life support [1, 2]. Because of the shortage of resources, the number of patients who can be followed and treated is limited and physicians should assign patients to critical care services in an appropriate and optimal way to increase the benefits of patient care, as well as improve patient safety [3–5]. On the other hand, patients have perplexing clinical manifestations which hinder reasonable assurance regarding treatment approaches and prognosis [6, 7]. Besides, delay or suboptimal care of severely ill patients may lead to increased mortality [8, 9].

Early identification of critically ill patients significantly impacts on patient's outcomes [5, 7, 10]. Scoring systems are based on physiological parameters [11–13]. Altered physiology, as reflected in aberrant vital signs and other findings, often precedes patient deterioration and death [14]. The objective information provided by these severity-of-illness scoring systems are considered a beneficial instrument tailored for supporting healthcare professionals to timely recognize and manage the critically ill patients who are at high risk of undesirable outcomes [15, 16].

A variety of scoring systems have been designed and commonly used for use in the emergency departments (EDs) such as WPS [17], REMS [18], and MEWS [19] and the intensive care unit (ICU) such as APACHE II [20], SAPS II [21], and SOFA [22] which are mostly based on vital signs and some laboratory results obtained within the first 24 h postadmission. The variables included in each scoring system plus their point assignment are presented in Table 1.

We can highlight a few aspects that exist in emergency models, in addition to simplicity, practicality, and good prognostic ability for the outcomes of interest. These models rely on a few numbers of variables which are easily available for all patients [14]. In contrast, ICU-based scoring systems include a greater number of factors that are frequently accessible only in severely ill patients [23, 24].

Although several studies have been performed for use in the EDs and ICUs, it is unknown which model is most suitable. Furthermore, there is no study that compares the ED models such as WPS, REMS, and MEWS with ICU models such as the APACHE II, SAPS II, and SOFA as ICU models in the ICU settings. So, the purpose of this study is to evaluate the performance of the WPS, REMS, and MEWS scoring systems in predicting the mortality rates of critically ill patients admitted to the ICUs.

## 2. Method

### 2.1. Study Design and Setting

**Figure 1:**
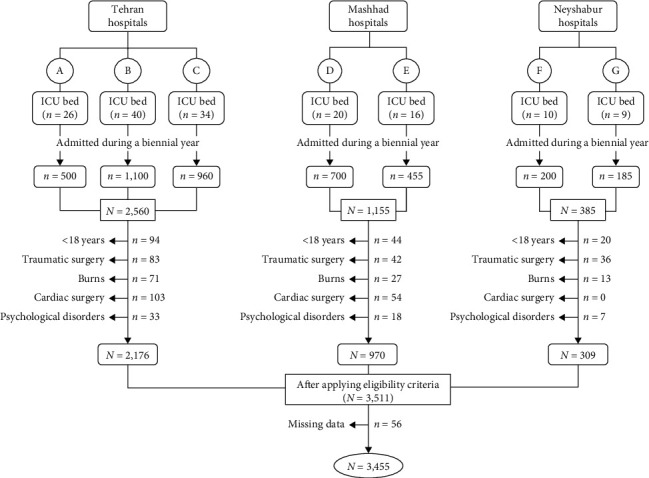
Schematic diagram of the inclusion/exclusion process. All ICU types are general/surgical.

An observational retrospective study was conducted to collect a prespecified set of variables in three referral centers in Tehran, the capital of Iran (three hospitals with 100 ICU beds), Mashhad in northeast Iran (two hospitals with 36 ICU beds), and Neyshabur in northeast Iran (two hospitals with 19 ICU beds). Because all of these seven centers are tertiary referral hospitals that serve a large portion of the population, they may be considered a sample of the entire population, with the results attributable to the community. More information about the participating hospitals and the distribution of the patients is presented in Figure 1. Because of the noninterventional design of the study, no informed consent was required.

### 2.2. Inclusion and Exclusion Criteria

We enrolled all critically ill adult patients (age ≥ 18 years) admitted to the ICU directly from the ED between August 2018 and August 2020. Those patients who were admitted due to traumatic surgery, burns, cardiac surgery, and psychological disorders were excluded due to the nature of the diagnoses [25]. In addition, any use of psychotropic agents in patients' medication profiles or symptoms of dysarthria or paramnesia (due to a type of brain disorder) were excluded similar to other studies in the field [20, 25]. Figure 1 illustrates the whole inclusion/exclusion process.

### 2.3. Data Collection

Structured forms including ICUs' models in addition to some variables used by the EDs' model were designed to be filled in for all included patients (*N* = 3,346). The highest physiological score for each particular patient during the first 24-hour period postadmission was considered the final score. The endpoint was defined as inhospital mortality regardless of the duration of the hospital stay (i.e., occurrence of death during an ICU stay or in another ward after ICU).

### 2.4. Statistical Analysis

Statistical analysis was performed using the R Statistical Software version 4.1.0. The packages pROC, Hmisc, rms, and Resource Selection were employed.

Continuous variables were expressed as a mean and standard deviation (SD). Categorical variables were expressed as number plus percentage. Between-group differences for quantitative and qualitative variables were assessed using the Student *t*-test and the Chi-squared test or Fisher's exact test. All tests were two-tailed. We also applied logistic regression to develop models including each scoring systems. The following formula was used to compute the expected probability for each individual patient:

(1) $$\begin{array}{c} P=\frac{1}{1+\exp\left[-\left(\beta_{0}+\beta_{1}X_{1}\right)\right]}, \end{array}$$

where *β*_0_ is the intercept; *β*_1_ is the coefficient of the score, and *X*_1_ is the score.

Validation of the ICU models and the ED models was assessed by discrimination, calibration, and accuracy of predicted probabilities.

Discrimination was measured using the area under the receiver operating characteristic curve (AUC-ROC) which is a measure of how much the model can distinguish between patients who have and do not have the outcome of interest (in our study, inhospital mortality). The exact binominal 95% confidence intervals (CI) for the AUROCs were also calculated. The differences between AUROCs were measured using the method proposed by DeLong et al. Diagnostic accuracy was defined as fail if an AUROC was 0.50-0.60, poor if an AUROC was 0.60-0.70, fair if an AUROC was 0.70-0.80, good if an AUROC was 0.80-0.90, and excellent if an AUROC was 0.9-1 [15]. The area under the precision-recall curve (AUPRC) was also used to inspect the trade-off between precision and recall as a measure of balance between the positive predictive value and sensitivity.

Calibration was assessed using calibration plots and the Hosmer-Lemeshow (HL) goodness-of-fit test. To generate smooth calibration plot, 1,000 bootstrap replicates were applied. The calibration plot is drawn by plotting the predicted probabilities on the *x*-axis and the actual probability of mortality, which represent the degree of concordance between the actual and predicted probabilities. To determine an optimal threshold value on the predicted probabilities, the Youden Index was used, and based on this threshold, we calculated sensitivity, specificity, positive predictive value (PPV), and the negative predictive values (NPV) for all models.

Accuracy of predicted probabilities was measured by the Brier score (BS) which is a quadratic scoring rule, where the squared differences between actual binary outcomes and predictions are calculated by the following formula: BS = (1/*N*)∑_*i*=1_^*N*^(predicted probabilty − actual outcome)^2^ [26].

The missing values were handled by taking into account the following consideration: patients with multiple laboratory and physiological missing values were excluded. The data of those patients with just one missing data were imputed by taking the value of the next day from their charts, and if this variable was not mentioned in the next day, it was considered normal.

We follow the TRIPOD (Transparent Reporting of a multivariable prediction model for Individual Prognosis or Diagnosis) statement for improving the transparency of reporting.

## 3. Results

The mean age of 3,455 included patients was 56.65 ± 21.52 years, and 1,879 (54.4%) males were covered in the study. Readmissions (*n* = 200) were excluded from the analysis. Only 60 eligible patients missed several laboratory or physiological parameters so they were excluded from the study. About 6 percent of patients' data (*N* = 204) was imputed by using the approach described in Method.

The overall inhospital mortality was 26.5% (916 out of 3,455 patients). The baseline characteristics of patients are presented in Table 2. The mean scores of the WPS, REMS, MEWS, APACHE II, SAPS II, and SOFA were 2.34 ± 1.65, 6.14 ± 3.41, 3.74 ± 2.30, 21.50 ± 6.74, 38.13 ± 14, and 3.19 ± 2.41 points, respectively.

**Figure 2:**
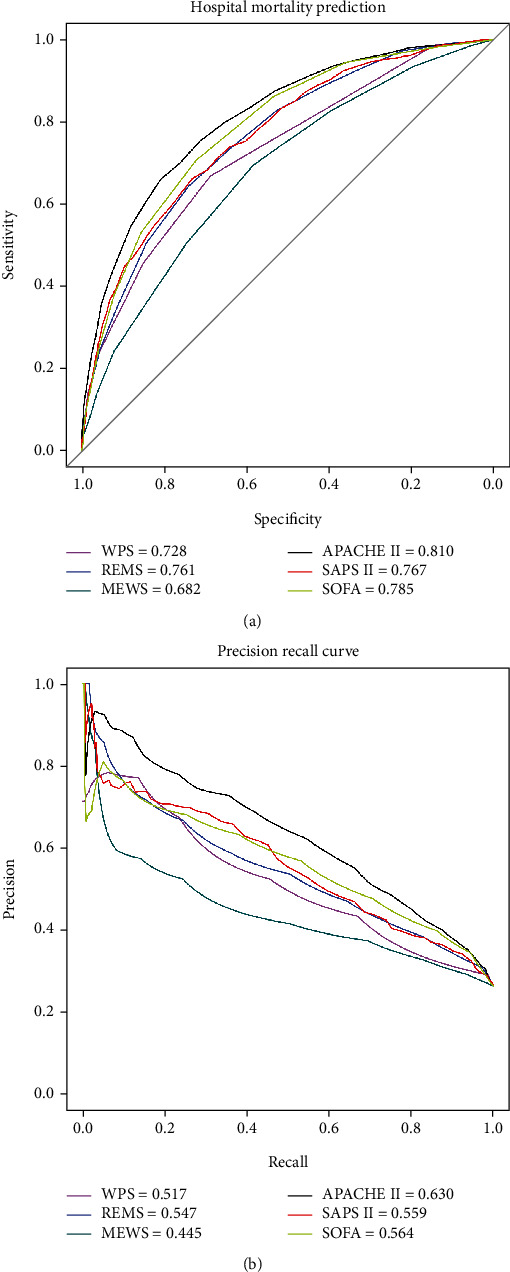
(a) The area under the precision-recall (PR) curve represents how a model balances the sensitivity and the positive predictive value. The *y*-axis represents the precision (positive predictive value in medical terms), and the *x*-axis represents recall (sensitivity). (b) The receiver operating characteristic (ROC) curves graphically represent sensitivity on the *y*-axis, and 1-specificity on the *x*-axis. The area under the curve (AUC) gauges the discriminatory ability of a model.

**Figure 3:**
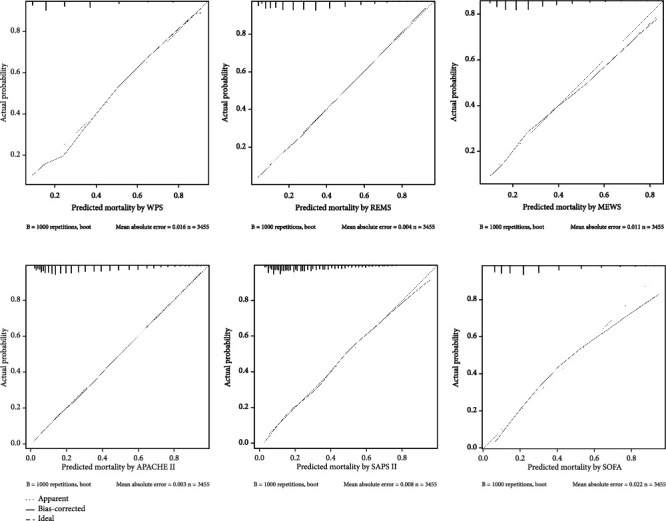
Calibration plots of the six models. A calibration plot is a measure of goodness-of-fit as a graphical presentation of the actual mortality probability versus the predicted mortality probability. The calibration plots of APACHE II, REMS, and SAPS II do not deviate much from the diagonal line, which represents perfect calibration.

Among the six investigated models, only the APACHE II predicted inhospital mortality with good discriminatory ability, while the WPS, REMS, SAPS II, and SOFA had fair discriminative ability and the MEWS had poor discriminative ability. The maximum AUPCR was also achieved by APAHCE II (0.63, see Figure 2). As shown in Figure 3, the APACHE II and its abbreviated version (the REMS) had no evidence of miscalibration (*p* = 0.9 for Hosmer-Lemeshow goodness-of-fit), whereas for the WPS and the SOFA, there is statistically significant evidence of miscalibration (*p* < 0.05), see also Table 3). As we present in Table 3, the best overall performance belongs to the APACHE II with the lowest Brier score (0.157), while the worst belongs to the MEWS with highest Brier score (0.183). The pairwise comparison of AUROCs is also presented in Table 4.

## 4. Discussion

The application of scoring systems in ICU has expanded dramatically for benchmarking and assessing quality of care [23]. In this study, we thoroughly assessed several scoring systems on discrimination, balance between sensitivity and the positive predictive value, calibration, and overall accuracy of the predicted probability.

### 4.1. Main Findings

We found that among all models examined, the APACHE II did not only have the highest discrimination ability but also had the best accuracy of the predicted probabilities, which was statistically significantly different from the other models in our setting. The mean predicted mortality by APACHE II (31.7%) was higher than the observed mortality (26.5%), and it is probably due to the care provided during the ICU stay and the quality of the follow-up care. The impressive APACHE performance in our cohort could be explained by the exclusion of trauma patients and patients with isolated neurological problems.

The APACHE II, REMS, and SAPS II indicated good agreement between actual and predicted probability of inhospital mortality throughout the whole range of predicted probabilities. In contrast, the SOFA and MEWS demonstrating their propensity to overestimate the inhospital mortality rate for the probabilities larger than 0.50 while the WPS underestimates it.

In this study, with the exception of the APACHE II and the MEWS which are at both ends of the good and poor spectrum, the other ICU and ED models are comparable.

### 4.2. Comparison to Other Similar Studies

Our findings is in line with a previous study [23] that indicated the fair discrimination power for the APACHE II and SAPS II (AUROC: 0.779 and 0.793, respectively). However, the discriminative ability of the REMS and MEWS was evaluated as virtually equal (AUROC: 0.738 and 0.729) in their study. Furthermore, although the discriminatory ability of the REMS and SAPS II was in the fair range, there was a significant difference between AUCs in that study [23]. The REMS and SAPS II had the equal AUCs in our study. Our findings are also consistent with another study showing higher discrimination of the APACHE II in prognostication than the SAPS II (AUROC: 0.828 vs. 0.782) [27]. Similar results were obtained in a study by Khwannimit and Geater, who compared the APACHE II and SAPS II [28]. Another investigation demonstrated that APACHE II, SAPS II, and SOFA had comparable high discriminatory ability [29].

Badrinath et al. reported that among various scoring systems applied on sepsis patients admitted to the ICU, the APAHCE II was more sensitive and specific in predicting mortality than the SOFA and REMS, which is in line with our findings. However, the discrimination power of APACHE II and REMS was evaluated as good and equal (AUROC: 0.81 vs. 0.80). This disparity was most likely caused by the patient population examined.

The APACHE and SOFA advantage is being able to be used to track a patient's response to therapy throughout their hospital stay. The APACHE II upon admission is around 75% accurate as an early prognostic indication of illness severity [30]. The better prognostic results obtained using the APACHE II score may be attributed to the additional physiological variables involved in calculating the APACHE II score. This may reflect the greater degree of organ dysfunction when calculating the APACHE II score as compared with other prognostic scores. Besides, the impressive APACHE performance in our cohort could be explained by the exclusion of trauma patients and patients with isolated neurological problems.

Interestingly, despite the fact that the SOFA is primarily designed for prognosis in sepsis patients, compared to the APACHE II and REMS, it performed poorly (AUROC: 0.74 (95% CI: 0.67–0.80)) [31].

In contrast with our findings, there are some studies which showed that there is no superiority of APACHE II over SAPS II and they both had fair discrimination and performed the same as each other [32].

In the retrospective study by Gök et al. which included critically ill patients admitted to ICU, the effectiveness and reliability of the WPS, REMS, and MEWS in predicting mortality were assessed and the results indicated the AUROC of the WPS was higher than the REMS and MEWS (0.769 vs. 0.70 and 0.711, respectively), whereas in our study, the REMS appeared with the ability to discriminate more [3].

### 4.3. Limitations and Strengths

To our knowledge, this is the first multicenter cohort study aimed at investigating and comparing three ICU and three ED models in mortality prediction in Iran. In addition, we had a multicenter large sample of patients and with a very low number and percentage of missing values. Collecting patient data from seven tertiary referral hospitals regarding similar population distribution may increase the generalizability of the results to a large subset of Iranian population.

Although the two-year sampling duration adjusts the effect of time-related confounding factors and may assure the inclusion of probable seasonal disorders, time and sample-related limitations remain as an inevitable factor. Removing missing data was another limitation in this study. However, this included only 1.6% of the data and could not have meaningfully affected the findings.

### 4.4. Implications

Our findings have important implications. The REMS and the SAPS II have a fair discrimination without a significant difference between the AUCs. However, the REMS has less complexity (smaller number of variables) compared to SAPS II, and its discriminative power was exactly the same as the SAPS II. Both models showed partially good calibration although overestimated mortality. Generally, it can be inferred that the REMS is more cost-effective and can be easily applied as a good alternative to the SAPS II in the detection of patients who are at high risk of deterioration. The REMS is also superior to the WPS in terms of discriminative power.

### 4.5. Future Studies

We suggest further evaluation of recalibrated versions of these prediction models on large samples of target population. Also, nonparametric models from statistical machine learning may help improve model performance. It is proposed that APACHE II be integrated into the electronic medical record system to enable real time predictions. Prospective studies could investigate the effect of incorporating these models in real-time decision support on mortality and other outcomes.

## 5. Conclusion

The APACHE II was found to be the most appropriate model in predicting inhospital mortality of patients in the ICU for all three performance dimensions (discrimination, calibration, and accuracy of predicted probabilities) in Iran. Except MEWS, the rest of the models have a fair discrimination and partially good calibration. Interestingly, although the REMS is less complicated than the SAPS II, both models performed similarly to each other. The findings emphasize the fact that clinicians should utilize this method as part of a larger clinical assessment to manage patients more effectively.

## Figures and Tables

**Table 1:** The point assignment scheme of each scoring system.

Emergency's scoring systems														

Model (min-max)	Temp (°C)	SBP (mmHg)	MAP (mm Hg)	RR (BPM)	Pulse (BMP)	GCS			AVPU			O_2_ sat (%)	Age (year)	
WPS (0-14)	≥35.3; 0 <35.3; 3	≥100; 0 ≤99; 2	NA	≤19; 0 20-21; 1 ≥22; 2	≤101; 0 ≥102; 1	NA			A; 0 Other; 3			96-100; 0 94-95; 1 92-93; 2 <92; 3	NA	
REMS (0-26)	NA	NA	70-109; 0 50-69; 2 110-129; 2 130-159; 3 ≤49; 4 ≥160; 4	12-24; 0 10-11; 1 25-34; 1 6-9; 2 35-49; 3 ≤5; 4 ≥50; 4	70-109; 0 50-69; 2 110-139; 2 40-54; 3 140-179; 3 ≤39; 4 ≥180; 4	≥14; 0 11-13; 1 8-10; 2 5-7; 3 ≤4; 4			NA			>89; 0 86-89; 1 75-85; 3 <75; 4	<45; 0 45-54; 2 55-64; 3 65-73; 5 ≥74; 6	
MEWS (0-19)	≤35; 2 35-38.4; 0 ≥38.5; 2	≤70; 3 71-80; 2 81-100; 1 101-199; 0 ≥200; 3	NA	<9; 2 9-14; 0 15-20; 1 21-29; 2 ≥30; 3	<40; 2 41-50; 1 51-100; 0 101-110; 1 111-129; 2 ≥130; 3	NA			Alert; 0 React to voice; 1 React to pain; 2 Unresponsive; 3			NA	NA	
ICU's scoring systems														

Model (min-max)	Temp (°C)	MAP (mmHg)	Pulse (BPM)	RR (BPM)	Oxygenation (paO_2_/FiO_2_)	GCS	HCO_3_ (mmHg)	K (mmol/L)	Na (mmol/L)	Cr (mg/dL)	HCT (%)	TLC (%)	pH	Age (year)
APACHE II (0-71)	≥41; 4 39-40.9; 3 38.5-38.9; 1 36-38.4; 0 34-35.9; 1 32-33.9; 2 30-31.9; 3 ≤29.9; 4	≥160; 4 130-159; 3 110-129; 2 70-109; 0 50-69; 2 ≤49; 4	≥100; 4 140-179; 3 110-139; 2 70-109; 0 50-69; 2 40-54; 3 ≤39; 4	≥50; 4 35-49; 3 25-34; 1 12-24; 0 10-11; 1 6-9; 2 ≤5; 4	≥500; 4 350-499; 3 200-349; 2 <200 & PO_2_>70; 0 PO_2_ 61-70; 1 PO_2_ 55-60; 3 PO_2_<55; 4	15-GCS	≥52; 4 41-51.9; 3 32-40.9; 1 22-31.9; 0 18-21.9; 2 15-17.9; 3 <15; 4	≥7; 4 6-6.9; 3 5.5-5.9; 1 3.5-5.4; 0 3-3.4; 1 2.5-2.9; 2 <2.5; 4	≥100; 4 160-179; 3 155-159; 2 150-154; 1 130-149; 0 120-129; 2 111-119; 3 ≤110; 4	≥3.5; 4 2-3.4; 3 1.5-1.9; 2 0.6-1.4; 0 <0.6; 2	≥60; 4 50-59.9; 2 46-49.9; 1 30.45.9; 0 20-29.9; 2 <20; 4	≥40; 4 20-39.9; 2 15-19.9; 1 3-14.9; 0 1-2.9; 2 <1; 4	7.7; 4 7.6-7.69; 3 7.5-7.59; 1 7.33-7.49; 0 7.25-7.32; 2 7.15-7.24; 3 <7.15; 4	<44; 0 45-54; 2 55-64; 3 65-74; 5 ≥75; 6
Model (min-max)	Temp (°C)	MAP (mmHg)	Pulse (BPM)	UO (mL/h)	Oxygenation paO_2_/FiO_2_	GCS	HCO_3_ (mmHg)	K (mmol/L)	Na (mmol/L)	Bili (mg/dL)	Chronic disease	TLC (%)	Urea (mg/dL)	Age (year)
SAPS II	<39; 0 >39; 3	<70; 13 70-99; 5 100-199; 0 >200; 2	<40; 11 40-69; 2 70-119; 0 120-159; 4 >160; 7	<500; 11 >500; 4 >1000; 0	<100; 11 100-199; 9 >200; 6	<6; 26 6-8; 13 9-10; 7 11-13; 5 14-15; 0	<15; 6 15-19; 3 >20; 0	<3; 3 3-4.9; 0 >5; 3	<125; 5 125-144; 0 >145; 1	<4; 0 4-5.9; 4 >6; 9	M.Cancer; 9 HemMalig; 10 AIDS; 17	<1; 12 1-20; 0 >20; 3	<28; 0 28-83; 6 >84; 10	<40; 0 40-59; 7 60-69; 12 70-74; 15 75-79; 16 ≥80; 8
	MAP (mmHg)		Oxygenation (paO2/FiO_2_)			GCS		Bili (mg/dL)		Cr (mg/dL)		Platelets (10 ^9^/L)		
SOFA	≥70; 0 <70; 1 Dop < 5 or Dob; 2 Dop 5.1-15 or Epi ≤ 1; 3 Dop > 15 or Epi > 0.1 or Nor > 0.1; 4		≥400; 0 <400; 1 <300; 2 <200+ RS; 3 <100+ RS; 4			15; 0 13-14; 1 10-12; 2 6-9; 3 <6; 4		<1.2; 0 1.2-1.9; 1 2-5.9; 2 6-11.9; 3 >12; 4		<1.2; 0 1.2-1.9; 1 2-3.4; 2 3.5-4.9; 3 >5; 4		≥150; 0 <150; 1 <100; 2 <50; 3 <20; 4		

Abbreviation: SCS: Simple Clinical Score; WPS: Worthing Physiological Scoring system; RAPS: Rapid Acute Physiology Score; REMS: Rapid Emergency Medicine Score; MEWS: Modified Early Warning Score; RLD: routine laboratory data; Temp: temperature; SBP: systolic blood pressure; MAP: mean arterial pressure; RR: respiratory rate; GCS: Glasgow Coma Scale; AVPU: alert, voice, pain, unresponsive; O_2_sat, oxygen saturation; Dop: Dopamine; Dob: dobutamine; Epi: epinephrine; Nor: norepinephrine; HR: heart rate; Bili: bilirubin; UO: urine output; HCT: hematocrit; Na: sodium; K: potassium; Cr: creatinine; BMP: beats per minute; BPM: breaths per minute.

**Table 2:** Baseline characteristics of study population.

Characteristics	Deceased (*N* = 915)	Recovered (*N* = 2540)	*P* value
Age (year)	66.27 ± 18.15	53.18 ± 21.59	<0.001^a^
Gender (male)	507 (55.4%)	1372 (54%)	0.511^b^
*Vital signs*			
Temperature (°C)	37.94 ± 15.68	37.42 ± 9.80	0.25^a^
MAP (mmHg)	98.13 ± 25.44	95.85 ± 16.09	0.012^a^
Pulse rate (beats/min)	96.72 ± 26.54	94.85 ± 23.24	0.06^a^
Respiratory rate (per min)	20.27 ± 10.73	17.71 ± 5.49	<0.001^a^
UO	1624.9 ± 833.2	1727 ± 880.3	0.002^a^
Oxygenation			
FiO_2_ (%)	24.31 ± 10.73	22.01 ± 7.96	<0.001^a^
O_2_ saturation	94.4 ± 3.30	96.8 ± 1.64	<0.001^a^
MV	77 (8.41%)	29 (1.14%)	<0.001^b^
Level of consciousness			
Glasgow Coma Scale (GCS)	8.68 ± 3.74	11.43 ± 3.32	<0.001^a^
AVPU			
(i) Alert	120 (13%)	979 (39%)	<0.001^c^
(ii) Voice responsive	174 (19%)	532 (21%)	
(iii) Pain responsive	248 (27%)	637 (25%)	
(iv) Unconscious	373 (41%)	392 (15%)	
Laboratory parameters			
Urea (mg/dL)	80.13 ± 71.32	70.02 ± 71.11	<0.001^a^
Creatinine (mg/dL)	2.17 ± 1.53	1.20 ± 1.10	<0.001^a^
Sodium (mEq/L)	139.9 ± 7.78	139.7 ± 20.44	0.78^a^
Potassium (mEq/L)	4.17 ± 0.83	4.08 ± 0.65	0.004^a^
Bilirubin (gr/dL)	1.15 ± 1	1.03 ± 0.84	0.002^a^
White blood cell (^∗^10^9^/L)	12.55 ± 6.37	11.99 ± 5.83	0.022^a^
Platelet (^∗^10^9^/L)	212.45 ± 121.9	235.36 ± 103.84	<0.001^a^
Hematocrit (gr/dL)	33.96 ± 7.31	34.84 ± 6.51	0.002^a^
PH	7.25 ± 0.44	7.32 ± 0.31	<0.001^a^
PaO_2_/FiO_2_	453.7 ± 147.67	470.7 ± 137.2	0.002
PaO_2_	104.46 ± 35.6	101 ± 30.5	0.009
HCO_3_	24.14 ± 8.31	26.27 ± 7.67	<0.001^a^
Comorbidities and other conditions			
DM	114 (12.4%)	42 (1.7%)	<0.001
Addiction	23 (2.51%)	14 (0.55%)	<0.001^b^
Metastatic cancer	19 (2.07%)	14 (0.55%)	<0.001^b^
ED risk scores			
WPS	3.39 ± 1.80	1.96 ± 1.41	<0.001^a^
REMS	8.46 ± 3.17	5.30 ± 3.09	<0.001^a^
MEWS	4.86 ± 2.50	3.33 ± 2.08	<0.001^a^
APACHE II	27.02 ± 6.34	19.50 ± 5.69	<0.001^a^
SAPS II	48.06 ± 14.33	34.53 ± 12.12	<0.001^a^
SOFA	5 ± 2.47	2.53 ± 2.01	<0.001^a^

Values are presented as mean ± SD or *N* (%). Abbreviations: ESI: Emergency Severity Index; FiO_2_: fraction of inspired oxygen; PCO_2_: partial pressure of carbon dioxide; HCO_3_: bicarbonate; MAP: mean arterial pressure; GCS: Glasgow Coma Scale; MV: mechanical ventilation; WPS: Worthing Physiological Score; REMS: Rapid Emergency Medicine Score; MEWS: Modified Early Warning Score; APACHE: Acute Physiologic and Chronic Health Evaluation (version II and IV); SAPS: Simplified Acute Physiology Score (version II); SOFA: Sequential Organ Failure Assessment. ^a^Analysis by independent-samples *t*-test. ^b^Analysis by Fisher's exact test. ^c^Analysis by Chi-square test.

**Table 3:** Intercept and slope of the linear predictor of the logistic regression for all models to predict inhospital mortality in ED, the optimism-corrected performance measures, and various threshold-based metrics (the threshold is itself based on the Youden index).

Models	Intercept (*β*_0_)	Slope (*β*_1_)	AUC-ROC (95% CI)	AUC-PR (95% CI)	BS	SE	HL	Threshold^∗^	Sen	Spe	PPV	NPV	Accuracy
WPS	-2.447	0.546	0.728 (0.71-0.75)	0.517 (0.50-0.53)	0.178	0.086	<0.05	2.5	0.669	0.686	0.435	0.851	0.682
REMS	-3.21	0.32	0.761 (0.74-0.78)	0.547 (0.53-0.56)	0.165	0.009	0.192	7.5	0.644	0.740	0.472	0.852	0.714
MEWS	-2.19	0.29	0.682 (0.66-0.70)	0.445 (0.42-0.46)	0.183	0.011	0.23	3.5	0.694	0.584	0.375	0.841	0.613
APACHE II	-5.691	0.201	0.810 (0.79-0.83)	0.630 (0.61-0.65)	0.157	0.008	0.9	24.5	0.661	0.807	0.553	0.868	0.768
SAPS II	-4.22	0.078	0.767 (0.75-0.79)	0.559 (0.54-0.58)	0.170	0.009	0.073	44.5	0.662	0.730	0.470	0.857	0.712
SOFA	-2.71	0.466	0.785 (0.77-0.80)	0.564 (0.54-0.57)	0.182	0.009	<0.05	3.5	0.706	0.722	0.478	0.872	0.718

Abbreviations: AUC-ROC: area under the receiver operating characteristic curve; AUC-PR: area under the precision-recall curve; CI: confidence interval; BS: Brier score; PPV: positive predictive value; SE: standard error; HL: Hosmer-Lemeshow; Sen: sensitivity; Spe: specificity; NPV: negative predictive value; SCS: Simple Clinical Score; WPS: Worthing Physiological Score; RAPS: Rapid Acute Physiology Score; REMS: Rapid Emergency Medicine Score; MEWS: Modified Early Warning Score; RLD: routine laboratory data; ED: emergency department. ^∗^This threshold is calculated based on the Youden index.

**Table 4:** Pairwise comparison of AUCs by using the DeLong test for each pair of model.

DeLong	WPS	REMS	MEWS	APACHE II	SAPS II	SOFA
WPS	∗	0.004	<0.001	<0.001	0.001	<0.001
REMS		∗	<0.001	<0.001	0.415	0.01
MEWS			∗	<0.001	<0.001	<0.001
APACHE II				∗	<0.001	0.001
SAPS II					∗	0.021
SOFA						∗

Abbreviations: SCS: Simple Clinical Score; WPS: Worthing Physiological Score; RAPS: Rapid Acute Physiology Score; REMS: Rapid Emergency Medicine Score; MEWS: Modified Early Warning Score; RLD: routine laboratory data.

## Data Availability

The datasets used and/or analyzed during the current study are available from the corresponding author (Dr. Saeid Eslami) at reasonable request.
